# The Development of Sugar-Based Anti-Melanogenic Agents

**DOI:** 10.3390/ijms17040583

**Published:** 2016-04-16

**Authors:** Bum-Ho Bin, Sung Tae Kim, Jinhyuk Bhin, Tae Ryong Lee, Eun-Gyung Cho

**Affiliations:** 1 AmorePacific Corporation Research & Deveolopment Center, Yongin, Gyeonggi-do 446-729, Korea; bbh82429@amorepacific.com (B.-H.B.); sungtae7@amorepacific.com (S.T.K.); 2 Department of Chemical Engineering, POSTECH, Pohang 790-784, Korea; bynjh007@postech.ac.kr

**Keywords:** sugar, melanin, anti-melanogenic agent, stress *N*-glycosylation, osmotic stress

## Abstract

The regulation of melanin production is important for managing skin darkness and hyperpigmentary disorders. Numerous anti-melanogenic agents that target tyrosinase activity/stability, melanosome maturation/transfer, or melanogenesis-related signaling pathways have been developed. As a rate-limiting enzyme in melanogenesis, tyrosinase has been the most attractive target, but tyrosinase-targeted treatments still pose serious potential risks, indicating the necessity of developing lower-risk anti-melanogenic agents. Sugars are ubiquitous natural compounds found in humans and other organisms. Here, we review the recent advances in research on the roles of sugars and sugar-related agents in melanogenesis and in the development of sugar-based anti-melanogenic agents. The proposed mechanisms of action of these agents include: (a) (natural sugars) disturbing proper melanosome maturation by inducing osmotic stress and inhibiting the PI3 kinase pathway and (b) (sugar derivatives) inhibiting tyrosinase maturation by blocking *N*-glycosylation. Finally, we propose an alternative strategy for developing anti-melanogenic sugars that theoretically reduce melanosomal pH by inhibiting a sucrose transporter and reduce tyrosinase activity by inhibiting copper incorporation into an active site. These studies provide evidence of the utility of sugar-based anti-melanogenic agents in managing skin darkness and curing pigmentary disorders and suggest a future direction for the development of physiologically favorable anti-melanogenic agents.

## 1. Introduction

Melanin, produced by melanocytes involving the cutaneous regulatory network for epidermal homeostasis [1,2], is important for responding to and protecting the skin from environmental stresses, such as ultraviolet radiation (UVR) [3,4,5]. Melanin is synthesized from the substrate l-tyrosine and the intermediate l-dihydroxyphenylalanine (l-DOPA) via an enzymatic cascade within the melanosome and serve as hormone-like regulators of melanocyte function by modulating tyrosinase activity and melanosome formation [6,7]. In contrast to normal melanin production, the abnormal activation of melanogenesis occurs through various extrinsic or intrinsic cues, e.g., repeated UV exposure, infectious/noninfectious biological factors, cutaneous pathological condition, or aging [8]. This cue-induced overproduction of melanin is triggered by cytokines and numerous hormones/neuropeptides, including corticotropin releasing hormone (CRH), proopiomelanocortin (POMC)-derived peptides (adrenocorticotropic hormone, α-melanocyte-stimulating hormone, β-endorphin), catecholamines, and acetylcholine via receptor-dependent and -independent pathways [7,9,10], leading to hyperpigmentation symptoms including uneven skin color, dark spots, and pigmentary disorders [11,12,13,14].

In the skin, factors affecting melanogenesis are secreted not only from skin cells, including keratinocytes and fibroblasts, but also from cutaneous immune, endothelial, and even nerve cells in conditions stimulated or stressed by UVR, indicating that the skin is a neuro-endocrine-immune organ [13,14]. The secreted factors bind to their specific receptors on melanocytes and lead to the stimulation of melanin synthesis via the cyclic adenosine monophosphate (cAMP)-dependent signaling, extracellular signal-regulated kinase (ERK) signaling, or Wnt/β-catenin signaling pathways [14,15]. These signaling pathways in turn modulate the expression or activity of microphthalmia-associated transcription factor (MITF), which is a master transcription factor that regulates the expression of the melanin-producing enzymes such as tyrosinase (TYR), tyrosinase-related protein 1 (TYRP1), and tyrosinase-related protein 2 (TYRP2) via binding to the M box of promoter regions of these genes. *De novo* melanin synthesis from l-tyrosine to eumelanin occurs through a sequential enzymatic reaction of these proteins within the melanosome [16,17]. Therefore, not only these enzymatic activities but also the formation, maturation, transport, and transfer of melanosomes are important factors for melanogenesis [13,14].

The development of anti-melanogenic agents is important for not only curing abnormal pigmentation for cosmetic, pharmaceutical, and medicinal purposes but also for melanoma therapy, in which melanogenesis and the pigment level can affect chemo-/radio-therapies and the survival period of patients with melanoma [18,19,20]. The components involved in *de novo* melanin synthesis, especially the rate-limiting enzyme TYR, have attracted much attention as regulatory targets for the development of anti-melanogenic agents in related fields. However, because these essential components are also implicated in physiological melanogenesis for maintaining cutaneous homeostasis and normal skin color, serious potential risks (*i.e.*, side effects) should be considered when targeting these molecules. Unfortunately, anti-melanogenic agents that target these molecules have often been accompanied by adverse effects, such as cytotoxicity and vitiligo-like symptoms [21,22]. For example, the recently approved 4-(4-hydroxyphenyl)-2-butanol (Rhododenol) competitively binds to the active site of TYR and inhibits its activity, leading to vitiligo-like symptoms that disappear after ceasing application [23,24].

This issue emphasizes the necessity of developing safer anti-melanogenic agents with fewer potential risks and adverse effects. Rather than directly targeting the components involved in melanin synthesis, as an alternative strategy to lower the risk of side effects, downregulation of the activation of melanogenesis-related intracellular signaling pathways has been suggested [25]. In addition, stimulation of the cutaneous melatoninergic system that protects against UV-induced oxidative stress has been considered as an anti-pigmentation treatment as well as a method to maintain skin integrity and homeostasis, suggesting that melatonin is an endogenous anti-melanogenic agent [26]. Considering that melanin is synthesized by a sequential enzymatic reaction of glycosylated enzymes, such as TYR, TYRP1, and TYRP2, within melanosomes and transferred to neighboring cells via melanosomes, the regulation of the glycosylation process for enzymes or the alteration of the melanosomal environment are other targets of hypopigmentation treatments. The protein stability of enzymes, and thus the activity of melanin synthesis, is dependent on glycosylation, and the melanosomal environment can be regulated by osmolality, pH, and ion concentration [27,28,29,30,31]. Thus, as natural ubiquitous compounds, sugars are worthy of study as possible anti-melanogenic agents without associated risks. In addition to its representative role as an energy source, sugar is implicated in various biological activities through the glycosylation of cellular proteins or lipids at defined sites via the enzyme-controlled addition of sugars [32] or glycation via a haphazard process. Likewise, sugars are involved in the translational modification of glycoproteins TYR, TYRP1, and TYRP2, either by becoming glycan units. As osmolytes, sugars can also induce hypertension pressure inside [33,34,35,36,37], which may affect melanosome formation, maturation, and trafficking.

In this review, we address the recent advances in research on the roles of sugars and sugar-related agents in melanogenesis, introducing their mechanisms of action [28,38,39,40] and the development of sugar-based anti-melanogenic agents. We further discuss an alternative strategy for the development of sugar-based agents that can be applied to human skin as anti-melanogenic agents.

## 2. Sugars as Anti-Melanogenic Agents

### 2.1. Natural Sugars

Sugars are widely used for cosmetic applications because of their moisturizing effects and relatively low cytotoxicity. Some sugars, such as neoagarobiose, have been shown to have comparable skin-whitening effects to other anti-melanogenic agents, including kojic acid and arbutin (~100 µg/mL) [41,42]. The possible use of sugars as anti-melanogenic agents has been discussed [43,44]; however, the mechanism of sugars in triggering hypopigmentation has not been clearly demonstrated.

Many tissues in our body encounter daily osmotic stress and adjust the osmotic pressure in various ways using osmolytes or osmolyte transporters [33,37,45]. As the outer covering of our body, skin encounters continuous osmotic stress, such as water transport and cell hydration, as it directly faces and borders the extrinsic environment; it is also affected by various intrinsic osmolytes [35,46,47]. Inside the epidermis, keratinocytes, major components of the skin, adapt themselves to osmotic changes by regulating the expression of organic osmolyte transporters along with changes in osmolytes [35]. Fibroblasts in the dermis also express these transporters, which are involved in antioxidant defense, protein stabilization, and stress responses to osmotic stress [34]. Similar to keratinocytes and fibroblasts, melanocytes located in the basement membrane between the epidermis and dermis are assumed to undergo osmotic stress, which may influence the main functions of melanocytes, such as melanin production, and may be a novel regulatory factor for melanogenesis.

Sugars have high solubility in water due to several hydroxyl residues in their structure, leading to high osmotic potentials, such as those of natural organic osmolytes, *i.e.*, sorbitol, inositol, and betaine [35,46,47]. Hyperosmotic stress induced by osmolytes significantly affects the biogenesis of intracellular cargo and lysosomal and endocytic compartments [48,49]. The presence of excessive organic osmolytes within cells induces the swelling of mannose 6-phosphate receptor (M6PR)-positive late endosomes as a consequence of endocytic membrane influx, which is coupled with the failure to transport cargo from the membrane to other intracellular destinations [48,49]. The melanosome is a lysosome-related organelle originating from the endosome; for melanin production, it should be properly matured from stage I to IV in a step-wise manner, which is largely dependent on organelle formation and the transport of melanogenesis-related proteins [50]. Therefore, osmotic stress induced by osmolytes, such as sugars, can directly influence melanosome properties and may become a novel regulatory factor for melanogenesis.

**Figure 1:**
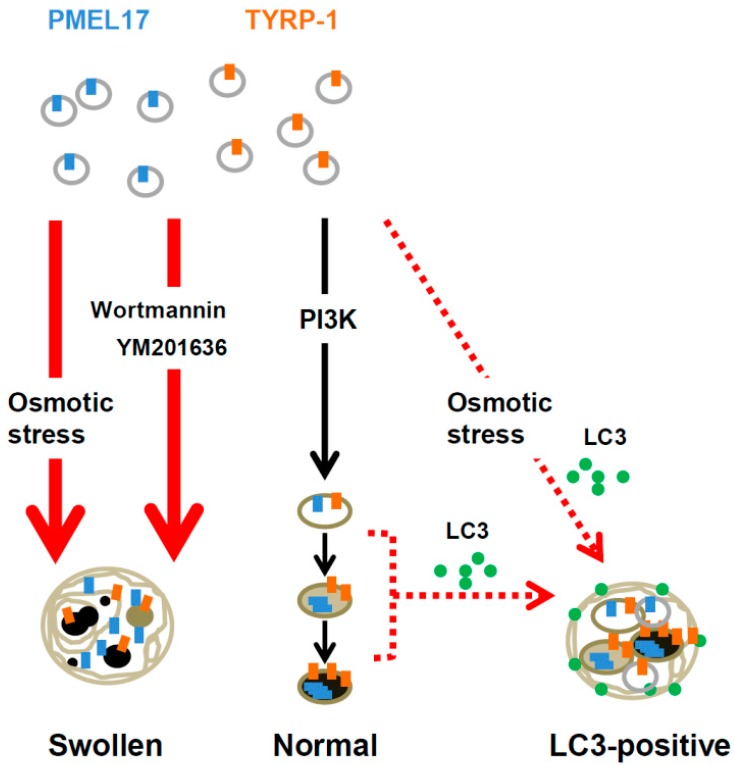
Osmotic stress disturbs normal melanosome formation by inhibiting the PI3K pathway. PI3K inhibitors, such as Wortmannin and YM201636, cause hypopigmentation in a similar manner to osmotic stress. Osmotic stress also induces the formation of LC3-positive melanosomes, but the possible involvement of these melanosomes in pigmentation remains unclear.

Consistent with previous reports on vesicle alteration by osmolytes [48,49], our study revealed that hyperosmotic stress induced by natural sugar treatment inhibits melanosome formation and maturation by inducing the swelling of M6PR-positive vacuoles and by inhibiting proper vesicle trafficking, resulting in hypopigmentation [28]. However, these vacuoles harbor the melanogenesis-related proteins TYRP-1 and PMEL17 (Figure 1) and display melanogenic activity when the substrate l-dihydroxyphenylalanine (l-DOPA) is exogenously supplied to cells, indicating that the swollen vacuoles are still capable of melanin synthesis but that the internal environment is likely to be incompatible with melanin synthesis [28]. Based on electron and confocal microscopy analyses, the morphology of abnormal melanosomes generated under hyperosmotic stress was highly similar to the morphology of vesicles generated through an impaired phosphoinositide 3-kinase (PI3K) pathway by PI3K inhibitors, such as Wortmannin and YM201636 [28]. The vesicles affected by hyperosmotic stress or by PI3K pathway impairment are M6PR-positive; therefore, the PI3K pathway is considered to have an important role in melanosome formation in melanocytes (Figure 1) [28]. Along with M6PR-positive abnormal melanosomes, hyperosmotic stress also induces autophagosome marker microtubule-associated protein 1A/1B-light chain 3 (LC3)-positive vacuoles (Figure 1) [28]. Given that there was no change in the expression level of melanogenesis-related proteins [28], which is expected in cases of a functional autophagosome, hyperosmotic stress does not likely induce the degradation of melanogenesis-related proteins or organelles via autophagy, leaving the possible role of LC3 in vacuole formation unknown. Of the disaccharides, sugars that are non-hydrolyzable by enzymes within human cells, such as sucrose and trehalose, showed more efficient inhibitory effects on melanosome formation than hydrolyzable sugars, such as maltose [28]. This result suggests that non-hydrolyzable natural sugars are potent inducers of hyperosmotic stress in melanocytes, leading to melanosome malformation through the disturbance of the PI3K pathway, and represent a new type of anti-melanogenic agent applicable for changing the melanosomal environment. 

### 2.2. Sugar Derivatives

The glycoprotein TYR, a rate-limiting enzyme in melanogenesis, catalyzes two steps in melanin biogenesis: (1) the hydroxylation of tyrosine to l-DOPA (tyrosine hydroxylase) and (2) the subsequent oxidation of l-DOPA to DOPA quinone (l-DOPA oxidase). For proper function and localization, TYR requires *N*-glycosylation at seven sites [30,51,52], and mutations at those sites are directly related to human oculocutaneous albinism (OCA), indicating the importance of this modification. *N*-glycosylation of TYR occurs step-by-step by the actions of enzymes, such as *N*-acetyl glucosaminyl transferases, in adding and enzymes, such as α-glucosidases and α-mannosidases, in detaching carbohydrates in the endoplasmic reticulum (ER) and Golgi apparatus [32,51]. If these enzymes are inhibited, TYR is aberrantly folded and undergoes degradation, resulting in hypopigmentation [51,52,53]. Hence, early attempts were made to use *N*-glycosylation inhibitors as anti-melanogenic agents [54,55]. Among general inhibitors of glycosylation, for example, glucosamine and tunicamycin effectively inhibit melanin production without decreasing the TYR protein level [56,57]. Two specific inhibitors, deoxynojirimycin (DNJ), an α-glucosidase inhibitor, and deoxymannojirimycin (DMJ), an α-mannosidase inhibitor, are also effective in decreasing melanin production [52,56]. Glutathione, ferritin, and feldamycin have also shown a strong inhibitory effect on melanin production [52,56]. However, the anti-melanogenic effects of glycosylation inhibitors are often accompanied by strong cytotoxic effects, such as cell cycle arrest.

Recently, we attempted to determine the mechanism of hypopigmentation by the anti-hyperglycemic agents acarbose, miglitol, and voglibose, which are pseudo-monosaccharide or -tetrasaccharide and are the most commonly used in clinics [40]. These anti-hyperglycemic agents possess inhibitory activity toward human α-glucosidase through incorporation into the active site, thus delaying the absorption of monosaccharides by inhibiting the digestion of carbohydrates in the small intestine [58]. The same inhibitory mechanism can be extended to intracellular glycosidases for the *N*-glycosylation of TYR. Based on our study in human melanocytes, of the three, voglibose exhibited the strongest inhibitory effect on melanin production by blocking the *N*-glycosylation of TYR but not of the other melanogenic enzymes TYRP-1 or -2, resulting in a dramatic reduction in TYR protein levels [40]. Because anti-hyperglycemic agents have already been used in clinical settings and do not trigger cytotoxicity in human melanocytes over a period, as demonstrated in our previous *in vitro* study [40], voglibose could be used as an anti-melanogenic agent with few side effects.

**Figure 2:**
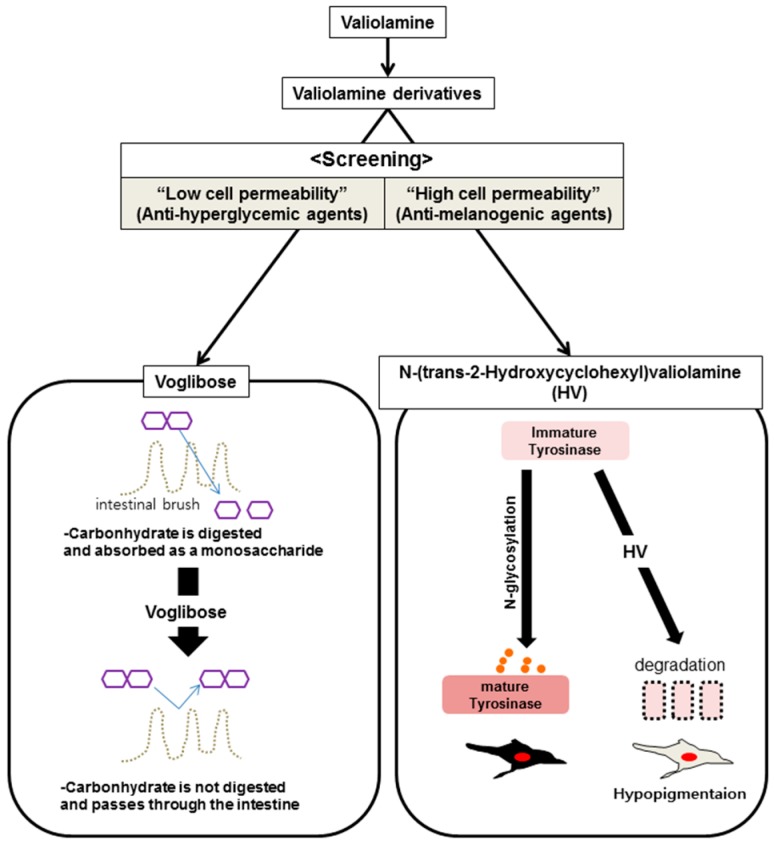
To develop an anti-hyperglycemic agent that works on the surface of the intestinal brush border, numerous valiolamine derivatives with low cell permeability were screened. In contrast, anti-melanogenic agents require high cell permeability to penetrate the skin barrier and influence melanocytes to inhibit *N*-glycosylation. An alternative screening method that depends on cell permeability could be used for screening for an effective anti-melanogenic agent, such as *N*-(*trans*-2-hydroxycyclohexyl)valiolamine (HV).

Voglibose synthesized from valiolamine was designed as a highly hydrophilic compound with hydroxyl groups, has a strong inhibitory effect on intestinal enzymes that digest carbohydrates, and has low cell-membrane permeability and high stability in the small intestine [58]. In contrast to inhibitors that target intestinal enzymes for digesting sugars, inhibitors that target intracellular glucosidases for the *N*-glycosylation of TYR in the endoplasmic reticulum (ER) or Golgi compartments of melanocytes require high cell permeability with relatively high hydrophobicity for better effects as anti-melanogenic agents. Therefore, in a previous study, we performed screening for anti-melanogenic agents with several N-substituted valiolamine derivatives synthesized to increase cell permeability (Figure 2) and displaying potent inhibitory activity toward intracellular glucosidases with no cytotoxicity. We discovered *N*-(*trans*-2-hydroxycyclohexyl)valiolamine (HV) and reported it to be a potent anti-melanogenic agent possessing higher anti-pigmentary activity than voglibose in a reconstituted human skin model [39]. Similar to the discovery of HV, with the goal of obtaining anti-melanogenic agents that are effective inside cells, N-substituted valiolamine derivatives can be alternatively designed and selected, according to the properties of target glycoproteins (Figure 2).

Among three major oral anti-hyperglycemic drugs, the pseudo-tetrasaccharide acarbose showed less anti-melanogenic activity compared with voglibose, although it had improved efficacy compared to that of voglibose [40]. We presumed that acarbose may be degraded by endogenous sugar-digesting enzymes, and thus hypothesized that acarbose could display more potent anti-melanogenic activity when co-treated with a hydrolysable sugar as a competing substrate. In our previous study, acarbose exhibited considerable anti-melanogenic activity when melanocytic cells were co-treated with acarbose and a digestible sugar, such as maltose (unpublished data) [59]. Simultaneous treatment with maltose augments the inhibitory effect of acarbose on α-glucosidase activity by enhancing the stability of acarbose under physiological conditions, leading to the downregulation of tyrosinase (unpublished data) [59]. Therefore, the co-treatment of anti-hyperglycemic agents with hydrolysable sugars may be a useful tool for reducing glucosidase-associated melanogenesis.

### 2.3. Membrane-Associated Transporter Protein (MATP): An Alternative Target for Anti-Melanogenic Sugars

**Figure 3:**
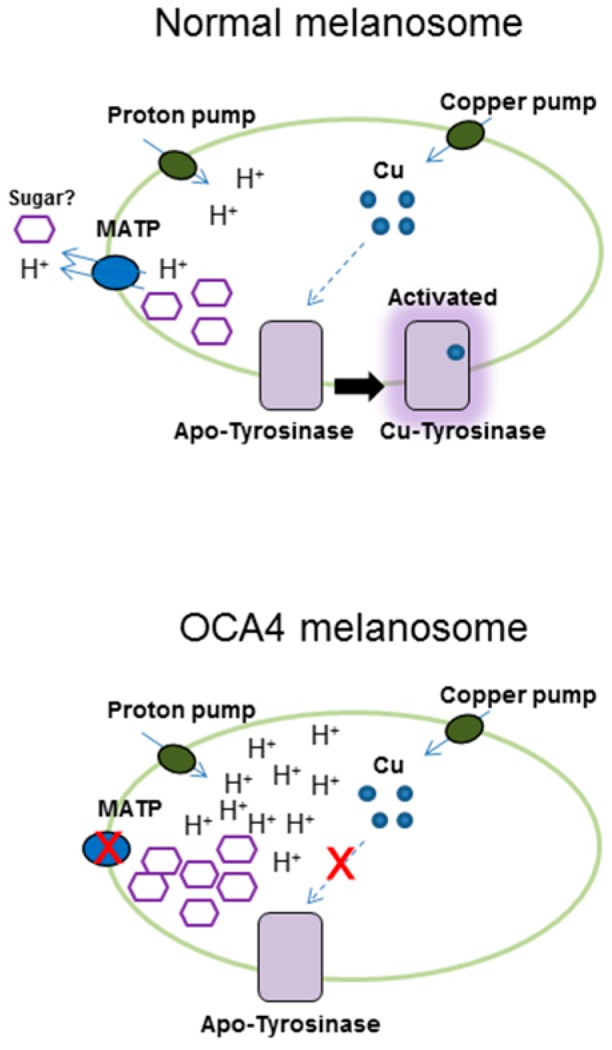
Membrane-associated transporter protein (MATP), a putative sucrose/H^+^ symporter, is present in the melanosome. MATP regulates melanosomal pH for copper binding in the active site of TYR. In the oculocutaneous albinism type 4 (OCA4) melanosome, mutations in MATP cause a loss of function, and the inner melanosomal pH becomes acidic, following the loss of copper ions from the active form of TYR. As a result, melanin production is downregulated. The arrows indicate the direction of substrates such as proton and copper.

A lack or reduction of melanin production in the skin and hair occurs frequently as a symptom of albinism, a hypopigmentation disorder [60]. Melanin deficiencies occurring in the eyes, hair and skin are called oculocutaneous albinism (OCA; types 1–4) and are associated with impaired vision and sunlight-sensitive skin [60,61]. Among the OCAs, OCA4 is mediated by a mutation in the *SLC45A2* gene, which encodes a sugar transporter-like membrane protein known as membrane-associated transporter protein (MATP) [62,63]. MATP is highly similar in amino acid sequence and protein structure to a functional sucrose transporter (SCRT) in *Drosophila melanogaster* [29] and to plant sugar uptake transporters (SUTs) [27,29] and possesses a typical sucrose transporter-specific motif (RxGRR) in its intracellular loop. Interestingly, in human cells, the *SLC45A2* gene of the SLC45 family is highly enriched in melanocytes and melanoma cell lines, and its protein, MATP, is located in melanosomes, implying a role for the sucrose transporter MATP in melanin synthesis [38]. As evidenced in our own and other studies [27,29,64], MATP transports sugar substrates via a proton-coupled transport mechanism, which is directly involved in maintaining the melanosomal pH in human melanocytes (Figure 3). Melanosomal pH is important for the proper activation of TYR, affecting the charged state of amino acid side chains in the active site and the binding of copper ions, which are essential co-factors for TYR activity [38,65,66]. The melanosomal pH is acidic at the immature stage of the melanosome and becomes alkaline during maturation, in which case, TYR is fully activated [67]. Based on our study, the reduced expression of MATP by siRNA or shRNA acidified the melanosomal pH more than in the control, leading to lower TYR activity [38]. As a sugar transporter, MATP possesses a sugar-binding site within its transporting pore, which may share structural similarity with the sugar-binding site present in glucosidases or other sugar-binding proteins [29,38]. Therefore, aside from downregulation of the MATP expression, as an alternative strategy to regulate melanosomal pH, substrate-mimicking inhibitory sugars or their derivatives could be designed to regulate MATP-transporting activity by comparing the pore site of MATP with those of transporters or active sites of enzymes that use sugars as substrates. So far, there is no available compound to regulate MATP activity. Additional biochemical characterization of MATP is necessary to determine its properties as a sugar transporter and to design inhibitory sugars to reduce the transporting activity of MATP. Considering that polymorphic mutations of MATP are clearly related to hypopigmentation without other defects [62,63] and the normal function of MATP is essential to melanosome physiology, sugar-based agents that inhibit MATP should be potently anti-melanogenic.

## 3. Conclusions

Few compounds can duplicate the broad role of sugar in living organisms. Herein, we have reviewed the recent advances in research regarding the roles of sugars or sugar-based agents in anti-melanogenesis and their mechanisms of action, *i.e.*, (1) the regulation of melanosome maturation by *natural sugars* via the induction of osmotic stress and (2) the inhibition of tyrosinase maturation by *sugar derivatives* via the inhibition of glucosidases for proper *N*-glycosylation. We described their utility as anti-melanogenic agents with or without further derivatization: the use of non-hydrolysable disaccharides for osmotic stress, the screening of valiolamine derivatives possessing higher cell permeability with anti-melanogenic activity, and the combination treatment of anti-hyperglycemic agents with other hydrolysable sugars. As an alternative target for the development of sugar-based anti-melanogenic agents, we suggest MATP, a sugar transporter. The MATP responsible for the hypopigmentary disorder OCA4 is highly relevant to pigmentation as a result of its effect on melanosomal pH, implying that sugar-based inhibitory agents of MATP activity could show potent anti-melanogenic activity with few side effects. In nature, sugars and their derivatives are widely used by various organisms for biological activities, such as germination, growth, and ripening in plants. Thus, understanding the roles of sugars and their derivatives and/or the sugar transport system in human melanocytes could provide novel insights not only for the development of physiologically favorable anti-melanogenic agents in the clinical and cosmetic fields in the future but also into the regulatory mechanisms of physiological activities.
